# Design of an online health-promoting community: negotiating user community needs with public health goals and service capabilities

**DOI:** 10.1186/1472-6963-13-258

**Published:** 2013-07-04

**Authors:** Joakim Ekberg, Toomas Timpka, Marianne Angbratt, Linda Frank, Anna-Maria Norén, Lena Hedin, Emelie Andersen, Elin A Gursky, Boel Andersson Gäre

**Affiliations:** 1 Department of Medical and Health Sciences, Linköping University, SE-581 83, Linköping, Sweden; 2 Centre for Public Health Sciences, Östergötland County Council, Linköping, Sweden; 3 Department of Health, Jönköping County Council, Jönköping, Sweden; 4 Centre for Public Health, Kalmar County Council, Oskarshamn, Sweden; 5 Analytic Services Inc, Arlington, VA, USA; 6 Futurum, Jönköping County Council, Jönköping, Sweden; 7 Jönköpings Academy for Improvement of Health and Welfare, Jönköping University, Jönköping, Sweden

**Keywords:** Community-based participatory research, Health promotion, Adolescents, Health service

## Abstract

**Background:**

An online health-promoting community (OHPC) has the potential to promote health and advance new means of dialogue between public health representatives and the general public. The aim of this study was to examine what aspects of an OHPC that are critical for satisfying the needs of the user community and public health goals and service capabilities.

**Methods:**

Community-based participatory research methods were used for data collection and analysis, and participatory design principles to develop a case study OHPC for adolescents. Qualitative data from adolescents on health appraisals and perspectives on health information were collected in a Swedish health service region and classified into categories of user health information exchange needs. A composite design rationale for the OHPC was completed by linking the identified user needs, user-derived requirements, and technical and organizational systems solutions. Conflicts between end-user requirements and organizational goals and resources were identified.

**Results:**

The most prominent health information needs were associated to food, exercise, and well-being. The assessment of the design rationale document and prototype in light of the regional public health goals and service capabilities showed that compromises were needed to resolve conflicts involving the management of organizational resources and responsibilities. The users wanted to discuss health issues with health experts having little time to set aside to the OHPC and it was unclear who should set the norms for the online discussions.

**Conclusions:**

OHPCs can be designed to satisfy both the needs of user communities and public health goals and service capabilities. Compromises are needed to resolve conflicts between users’ needs to discuss health issues with domain experts and the management of resources and responsibilities in public health organizations.

## Background

Health promotion denotes a set of principles for endorsement of the health of the population from an inclusive perspective. These principles differ from corresponding approaches for disease prevention, where epidemiological knowledge of specific health risks is used for prevention and early detection of particular diseases. The theoretical basis for health promotion is that health is determined in interaction with communities and their residents, and that changes in the physical and social living environment influence the health status [1]. Health is thus seen not only to reflect the absence of disease but also encompasses aspects such as capacity to fulfil vital life goals [2] and psychological well-being and outlook [3].

Due to the advances in web technology, Internet-based applications are now inexpensive alternatives for health promotion, e.g. by self-directed and informal learning in online communities. An online community refers to a gathering of individuals in a virtual space who form a network for long-term public discussions on common interests or experiences [4]. In contrast to geographic communities who share interests in physical tasks and activities, the emphasis in online communities is on learning and information exchange [5,6]. Self-directed learning, where the individual is in command of what should be learned, stands in contrast to health education, where health experts provide information that they think the recipients need [7].

An online health-promoting community (OHPC) has the potential to promote health and advance new means of dialogue between public health representatives and the general public. In Sweden more than 90% of adolescents have Internet access and go online several times a week [8]. However, an OHPC must be managed and even if advances in web technologies simplify the initiation of online communities, management and maintenance must be sustainable. An online community involving the health care service needs to be in accordance with public health goals and service capacity. Public health representatives cannot be expected to invest time and funding in an intervention at odds with the goals of the public health service.

In Sweden, obesity among children has not yet matched the epidemic proportions reported from other parts of the world [9-12]. However, among adolescents, overweight and self-consciousness regarding body shape, diet and exercise influence their social, psychological and physical health [13-15]. Obese children may be in need of secondary prevention because of the adverse effects related to obesity, such as development of coronary heart disease, related to long-term obesity [16], but it is less obvious what to prevent in the remaining population. General interventions, such as health education, to prevent overweight and obesity are problematic because of the lack of procedures that have general application [13,17]. There is a need for personalized community-based health promotion [18]. Online interventions are especially suitable for this purpose when considering the amount of time adolescents spend online [19].

The aim of this study was to examine what aspects of an OHPC that are critical for satisfying the needs of the user community and public health goals and service capabilities. The study was performed in a health care region (population 1 200 000) in the south east of Sweden, where analyses of obesity data had shown that the need for obesity prevention among adolescents was greater than the resources available [20]. The results indicated that obese adolescents would benefit from more specialized interventions; a need for an online public health intervention addressing the non-obese adolescent population was also identified.

## Methods

Community-based participatory research methods [21,22] were used for the study and participatory design principles were used for the OHPC systems development [23]. Community-based participatory research methods provide an arrangement for balanced influence and control over research processes by non-academic researchers and support structures and processes for academic–community partnerships to improve health [24].

**Figure 1:**
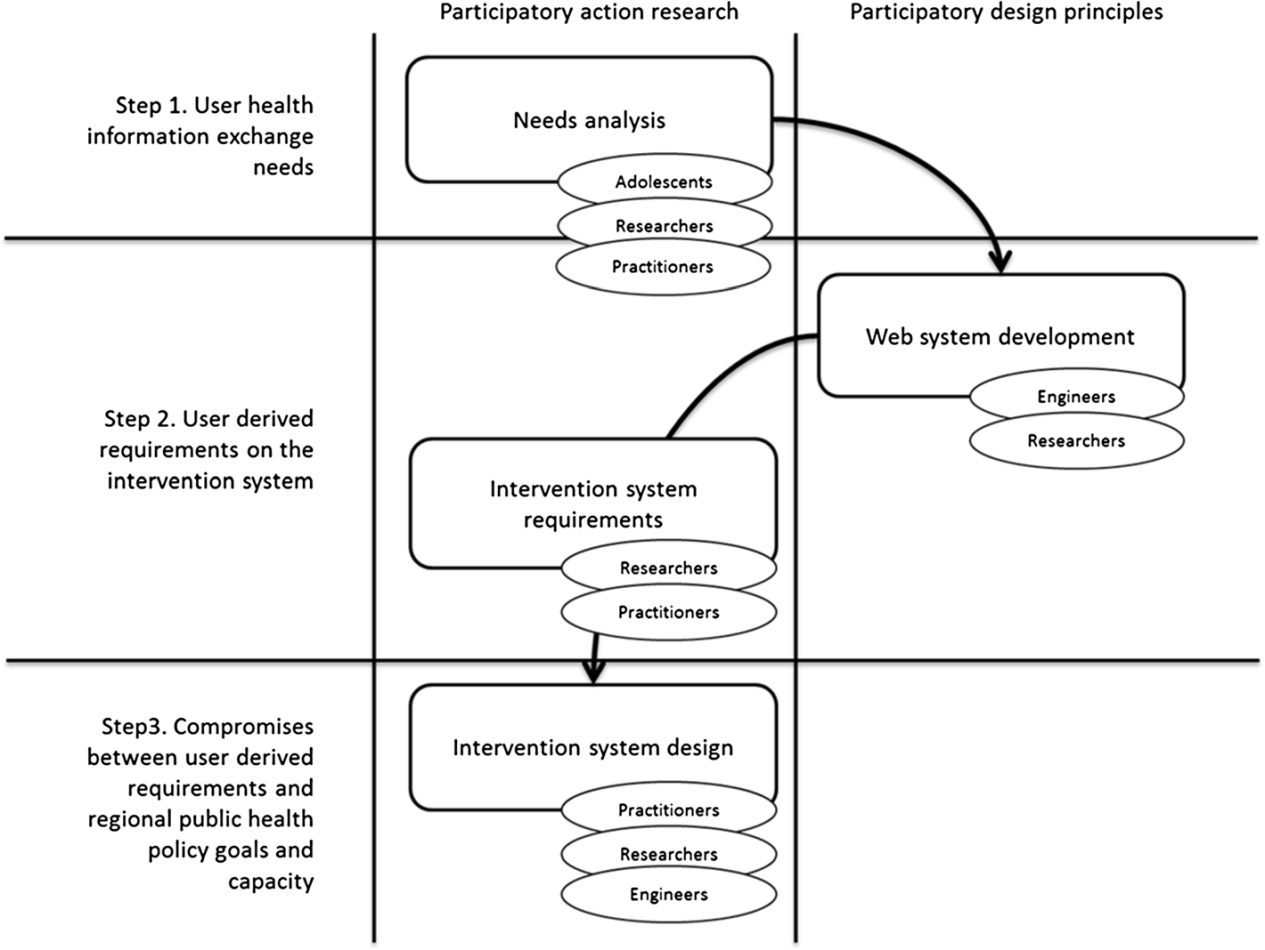
A three-step analysis to specify the structure and functions of an OHPC intervention.

Qualitative data from adolescents on health appraisals and perspectives on health information were collected in a Swedish health service region. Using a three-step analysis, the data were transformed to specify the structure and functions of an OHPC intervention (Figure 1). In the first step, the crude data were analysed and structured into health information needs. The identified needs were then transformed into requirements for an OHPC intervention by an interdisciplinary expert team. In this context, needs refer to problems expressed that warrant a solution; requirements are associated with the design of an information system [25]. In the third step, a prototype based on the user-derived requirements was compared with regional public health goals and service capabilities by public health official representatives from the region, and modified accordingly. Conflicts between the system requirements derived from the adolescents and public health goals and resources were identified.

### Data collection

The model population for the development of the OHPC intervention consisted of high school students in their senior year (17–18 years of age). A convenience sample (*n* = 65) representing the population of adolescents aged 15–20 years (*n* ≈ 50 000) in the study region was recruited. Focus group interview sessions were arranged with adolescents according to two criteria: home community sociocultural profile (industrial–technological, small business–church dominated, small business–agricultural) and high school program profile (high school program in science, arts or nursing). High schools corresponding to the listed program profiles in each study community were contacted and asked for assistance with recruiting students for focus group meetings. Qualitative data were collected using two sets of focus group interviews. The topics for the first set of interviews were physical health, psychological health, and health products; the second set of interviews covered health information in the media, information needs and information-seeking behaviour. The protocol for the focus group sessions is provided in Additional file 1. Initially, each student was asked to participate in both focus groups but some cancellations occurred and stand-ins were accepted. Of the 65 participating adolescents, 48 participated in both sets of focus group interviews, 12 only in the first, and 5 only in the second. In total 18 focus group sessions took place.

Data from the focus groups were collected over a 2-month period in 2010. The focus groups were video recorded and lasted for approximately 90 minutes for the first occasion and 60 minutes for the second. In total, approximately 20 hours of video were collected. In each focus group, two moderators were present: one moderating the discussion and the other making observations. The data were collected by means of formal natural group discussion defined as a group interview with invited people who already know each other [26]. The resulting requirements and various prototypes were demonstrated and discussed with representatives from regional public health services in meetings and telephone conferences.

The focus groups took place after hours in the students’ free time; informed consent was obtained from the participants and with the permission of schools and teachers. The project was approved by the Ethics Committee of Linköping University, Dnr: 479–31.

### Data analysis

#### Step 1. Identification of health information exchange needs

An analysis of health information exchange needs was performed using data from the focus group meetings. Summaries from each focus group were compiled as transcripts from the video recordings by one of the moderators and sent to the second moderator of the focus group to make sure that the summary was accurate. The transcribed content was coded based on themes identified in the material by the first moderator.

Statements from the focus groups related to health information exchange were identified and a summative content analysis of these units was performed to identify specific user needs as related to an information system. In addition, statements regarding content and topics of interest were used to assist in drafting the content for the information system.

#### Step 2. User-derived requirements for the information system

A process to determine the user-derived system requirements was undertaken based on the principles of design space analysis [27,28] with design rationales captured using an informal representation [29]. A design team consisting of two computer scientists, an interaction designer, and a cognitive scientist drafted the requirements for an OHPC based on the health information exchange needs identified. The design decisions were then constructed into a composite design rationale. A design rationale is an explicit list of decisions made during a design process, and the reasons why those decisions were made [30]. The design rationale primarily consisted of the identified user needs, the user-derived requirements, technical and organizational system solutions, alternative solutions, and the reason for their rejection when applicable. The design rationale was also presented as a prototype OHPC.

#### Step 3. Conflicts between user-derived requirements and regional public health goals and service capability

In the final step, the design rationale document and prototype were presented to a panel of six public health professionals working with adolescent obesity, representing the health service providers in the region. The task presented to the panel was to assess the prototype with regard to the regional public health goals and service capability for an OHPC intervention. Design solutions were reviewed in sessions at which the expected positive and negative consequences of each design solution were examined in light of its hypothetical implementation in the study setting. When conflicts between requirements, public health goals, and service capabilities were found, the solutions were adjusted in order to neutralize the conflicts. These adaptations were compiled, analysed for common features, and documented.

## Results

Step 1. Identification of health information exchange needs

All participants studied at their senior high school year (17–18 years of age), of which 69% were female. The focus group transcript was divided into 505 statement units related to health information exchange. These units were classified into ten categories of user health information exchange needs (Table 1); content-related needs were excluded. The most prominent content-related statements were about food, exercise, and well-being.

**Table 1:** User health information exchange needs

**Focus group context**	**User health information exchange need**
The adolescents exhibited impressive knowledge and had no shortage of simple health advice; they rather challenged traditional tenets of diet and exercise, not because they distrusted health advice, but because they distrusted everything. They did not lack information, but rather a method to discern the true from the false. Since almost everything health-related they know of is presented with the agenda of selling them a product, their rule of thumb was to generally disregard everything	The experience should be educational
Even though the adolescents disregarded information provided by health care organizations, they did believe that physicians and nurses could provide genuine useful advice. However, they also wanted a personal comment regarding their questions, not general advice addressing everyone	Personal advice from health professionals
Exploring subjects and seeing the point of view of others in discussion forums and boards was both appreciated and utilized among the adolescents; however, they had experienced saturation where the discussion was halted either because of lack of experts with more knowledge to inject into the discussion, or where a debate turned sour ending without any means to verify or check the validity of the claims given by either party	Discussions in which experts participate
The adolescents belong to a generation where most of their information sources are online and funded by ads, and where additional pages, reloads, and links increase the revenue of the website. It therefore was reported to be both a tedious and confusing task to access the content sought after	Easy accessible content
Most health-related information provided online was reported to be both abstract and general, or specific but provided to sell products. This included information from governmental sources and information from health care providers. There was reported a lack of information regarding the subjects they cared about and wanted to know more about	Information about our interests
Reading about faulty but widely held beliefs was seen as entertaining and informative, and the adolescents were fully aware that commercial interests both skewed the facts to push products, and also outright lied if they could get away with it. Having health professionals call this out was reported to be an exciting prospect	Dismantling of myths and misconceptions
Diets and types of exercise was an area where it was seen as very difficult to find useful information. Information from commercial interests was entirely distrusted, and since both diet and types of exercise are not only a comparison of efficiency, but also experience, sharing experiences among themselves was seen as valuable	Be able to share tips and experiences
The aesthetics of websites was reported to be used as a tool to determine the underlying agenda, trustworthiness, and target group of the website. In this process, very attractive sites could be immediately dismissed because it was apparent that such sites were drafted to push products. A clean, simple, professional site with a clear manifesto and clear agenda was seen as important for credibility among the adolescents	Professional and serious
One critique of the communication from the scientific community and health care providers was its reliance on a wall of text. Adolescents are sometimes accused of lacking attention span and in need of simple accessible short bursts of information. However, the adolescents reported that they were so overwhelmed by information that it has become necessary to be able to use heuristics to determine gold from grit. There was no aversion to in-depth information; rather there is a need to have very concise and simple introductions	Concise presentation
Even good communities were reported to be shunned because of bad manners and uncivil conduct. Moreover, it was emphasized that once a community gets a certain vibe, it sticks forever	Maintain a civil discourse

Step 2. User-derived requirements for the information system

The structures and functions identified as requirements to satisfy the adolescents’ health information exchange needs included both technical and organizational components. A composite design rationale for the OHPC was completed by linking the identified user needs, user-derived requirements, and technical and organizational system solutions. When applicable, alternative solutions and the reason for their rejection were also listed (Table 2).

**Table 2:** Reconstructed design rationales

**Adolescent user need**	**Adolescent user-derived requirement**	**Design solution**		**Alternative solutions and reasons for rejection**
		**Technical system**	**Organizational system**	
The experience should be educational	The intervention must be able to facilitate self-directed learning	Online discussion forum where the system aids in keeping track of new posts in areas of interest, requiring a user registration and user management system	–	Text/video chat, wiki, podcast/vodcasts, and blogs all violated a combination of other needs such as professional and serious, concise presentation and civil discourse
Personal advice from health professionals	Participation by health professionals who can be accessed	An online system for presenting experts and the ability to ask and get answers from experts	An expert panel representing the key public health areas available for regular questioning	
Discussions in which experts participate	Active participation by experts in a forum	An online discussion forum. Distinguishable user groups with different levels of authority	An expert panel that is willing to publicly engage in online discussions	A chat setup would satisfy this requirement, however there is less opportunity to satisfy the need of civil discourse and the need to exchange tips and experiences would be severely hampered
Information about our interests	User driven content	Reasonably accessible and permanent record of online discussions	Editorial content based on online discussion surveillance	
Easy accessible content	Simple categorical organization and presentation of content	Organization and presentation of content into a minimal set of categories. Content management system for categorization and presentation	-	
Dismantling of myths and misconceptions	Provide commentary on prevalent myths and misconceptions	Provision of intuitive methods for production of editorial material	Provision of editorial content by health care professionals based on current events and health-related media reports	
Be able to share tips and experiences	Provide means for users to post content	An online forum with registered user id with suitable categories. A user registration and management system	–	User blogs. The interest in blogging was assessed to be low, and problems of regulating what was off-topic made this undesirable
Professional and serious	A clean and minimalistic layout free from commercial interests	A content management system without ads. Design of an appropriate layout	Maintenance of layout integrity and content management	Maintenance of active communities without a content management system is prone to errors and mistakes in updates and layouts. A free and simple content management system with extensive community support dramatically reduces the load of maintenance
Concise presentation	Conform content to blogesque (short and sweet) format	A system to post videos and organize short articles	Utilize blogesque newsfeed and short video interviews	Editorial work on content; however, the work load involved was deemed to be high
Maintain a civil discourse	Vigilant moderation	Provide means for moderation of discussions	Enough moderators to keep up with online activity	Use of strict rules of conduct to educate users was discussed, but there will always be abuse online

Step 3. Conflicts between the user-derived requirements and regional public health goals and service capability

The assessment of the design rationale document and prototype in light of the regional public health goals and service capabilities raised concerns about organizational aspects of the design solutions. No technical aspects of the design solution were found to be problematic. In brief, compromises were needed to resolve conflicts involving the management of organizational resources and responsibilities.

Two conflicts arose about making a health panel available for questions from adolescents and engaging practitioners in online discussions to meet the adolescents’ needs (*personal advice from health professionals* and *discussions in which experts participate*). With regard to the resources at hand, monitoring the potentially vast number of questions and discussions was deemed to be too high a workload for the practitioners. The compromise found was to use a previously rejected design solution of using vodcasts. To produce the vodcasts, it was decided to appoint a community manager to monitor the discussion, collect questions and hot topics, and regularly interview the practitioners. A similar problem occurred when the practitioners were expected to produce editorial content as comments on current health-related events and media reports to meet the need for *dismantling of myths and misconceptions*. Because of the need for special care when producing material for the public from health services, these editorials were deemed to demand too much work. The compromise found was to transfer this responsibility to public health academics because they are more familiar with producing editorial material, and public communications from them would not be seen to come from the regional health service.

The fourth conflict was on the choice of OHPC editorial content based on monitoring of online discussions in order to meet the adolescents’ need for *information about our interests*. It was speculated that the editorial content would then be focused exclusively on topics with low priority in public health policies, e.g. make-up and skincare products. However, the practitioners were sympathetic to an approach to adapt the editorial content to adolescents’ interests and a compromise was decided whereby monitoring was to be accompanied by a clear statement of the purpose and focus of the OHPC.

The fifth conflict was about the moderators keeping up with the online activity if the OHPC intervention was used extensively, regarding the need to *maintain a civil discourse*. The compromise agreed on involved recruiting moderators from members of the adolescent user community based on exemplary conduct.

## Discussion

This study set out to examine what aspects of an OHPC that are critical for satisfying the needs of user communities and public health goals and service capabilities. The most important observation was that compromises in the OHPC design were needed to resolve conflicts between needs to discuss health issues with domain experts in the end-user population and resources and responsibilities related to online activities in the public health organization. In a comparable OHPC development initiative in Ontario, Canada (http://www.youthspark.ca), a method for systems development and evaluation of an OHPC has been comprehensively documented [31]. The current results add knowledge about the need to negotiate public health interests and end-user needs in the OHPC design process.

Web technology is becoming increasingly prevalent in health services [32,33]. In a recent review of computer and web-based interventions to increase physical activity among adolescents, most reported interventions had led to a significant increase in physical activity, but the effects were short lived [34]. Experiences from smoking cessation programs suggest that using only Internet initiatives based on social cognitive theory is not effective [35], but that a combination of cell phone-based “push” technologies, peer-to-peer contact, and web-based information on health and coping strategies can be a promising long-term treatment option [36]. In parallel, online communities providing several of the latter services to users have been used in health services for self-management of health problems [37] and establishment of communities of practice [38]. An OHPC can be regarded as a community-based, long-term, and inclusive health promotion program [1], although mediated over the Internet. When such OHPCs are developed on a large scale, the existing models for health promotion program implementation and evaluation must also be extended to fit the virtual setting. The OHPCs have to be evaluated with regard to behavioural patterns and health effects in the intended population. In these evaluations, there is thus a need to measure the outreach, usage, and effectiveness of the online system, using methods ranging from web surveys and focus groups to epidemiological studies. Such large-scale evaluations of OHPC designs in practice settings are much warranted, but also associated with considerable methodological challenges.

Participatory design proponents suggest that because social factors important in the implementation process are highlighted in a participatory approach, the resulting system designs are more sustainable compared with those developed using traditional systems development methods [39]. However, participatory methods have also been criticized for neglecting the later technical development stages [40] and being unrealistic with regard to the time investments demanded by practitioners [41]. Techniques such as the Voice of the Customer table and House of Quality [42] are well-established but resource-consuming approaches to the technical system accurateness problem. For instance, a possible problem with an OHPC initiative is that it may encourage young people to post and discuss health issues and problems in a manner that they later may come to regret. Even though it is easy to censor content after the fact, information online tends to persist. The use of design rationales in this study enabled public health practitioners to document this design problem, and analyse their views on the consequences of possible solutions. However, these views needed to be scrutinized also by engineers and legal expert before being implemented. This example illustrates that a participatory design process must be structured to include not only a representative group of end-users, but also the necessary expertise. If the experts not are available in the primary process, they have to be recruited downstream to adjust and approve the final systems design. For less complicated design problems, consultation of the scientific literature may be satisfactory. For the example problem, it is possible that consultation review of privacy guidelines for social media and maturation of online behaviour may have been sufficient to reduce the risks associated with sharing online [43].

The study has several limitations that need to be taken into account when interpreting the results. Allowing practitioners and the target community to influence the qualitative analysis process may be interpreted as surrendering control of the study. However, in accordance with the suggested increased use of qualitative data from in-depth interviews and observations for evaluating the context, process, impact, and outcome of community-based interventions [21,44-48], we contend that the participatory method used in this study is adequate for capturing the context and process of the community development. In such interventions, experimental control may be incongruous when taking the different sociopolitical structures of communities into account [44,46,49]. To ensure contextual soundness, the practitioners were in this study included in their actual professional roles, and the adolescents were regarded as the primary end-users, rather than merely informants. Also, including adolescents from the end-user community from several municipalities and school programs in the research was an attempt to decrease the threats to soundness that are associated with a convenience sample and that the adolescents would be disingenuous.

## Conclusions

The study shows that an OHPC can be designed to satisfy both the needs of the user community and public health goals and service capabilities. The main results are that compromises in OHPC design are needed to resolve conflicts between needs and requirements to discuss health issues with domain experts in the end-user population and the management of resources and responsibilities related to online activities in the public health organization.

## Abbreviations

OHPC: Online health-promoting community.

## Competing interests

The authors declare that they have no competing interests.

## Authors’ contributions

JE participated in the design of the study, performed the analysis and drafted the manuscript. TT participated in the design of the study, performed the analysis and drafted the manuscript. MA participated in the design of the study, performed the analysis and revised the manuscript. LF participated in the design of the study, performed the analysis and revised the manuscript. AN participated in the design of the study, performed the analysis and revised the manuscript. LH participated in the design of the study, performed the analysis and revised the manuscript. EA participated in the design of the study, performed the analysis and revised the manuscript. EAG participated in the design of the study, performed the analysis and revised the manuscript. BA participated in the design of the study, performed the analysis and revised the manuscript. All authors read and approved the final manuscript.

## Pre-publication history

The pre-publication history for this paper can be accessed here:

http://www.biomedcentral.com/1472-6963/13/258/prepub

## Supplementary Material

Additional file 1 Protocol for the focus group sessions. Click here for file
