# Supplementary material for: Design of an online health-promoting community: negotiating user community needs with public health goals and service capabilities
Source: BMC Health Serv Res. 2013 Jul 4;13:258. doi: 10.1186/1472-6963-13-258 (PMC3708753; doi:10.1186/1472-6963-13-258)
Supplement: Additional file 1 — Protocol for the focus group sessions. [file 1472-6963-13-258-S1.doc]

# Additional file 1 – Protocol for the focus group sessions

**Focus Group 1:** Health issues in adolescents’ lives

**Physical health (stamina, be strong, look good)**

**[Knowledge] What is physical health?**

What do you think, read and talk about regarding "health"?

**[Behaviour] What do you do to feel good?**

Food (meals, what kind of food), training (school, leisure, at all) and other things?

**[Attitudes] What do you think about diet and exercise?**

Slimming, diets, additives, nutrition, fitness, sport, exercise amount and type

**Mental health (feel good, be content)**

**[Knowledge] What is mental health?**

What do you think about, read about and talk about touching to feel good mentally

**[Behaviour] What are you doing to feel good?**

Sleep, relaxation, other?

**[Attitudes] What do you think of activities to "promote" well-being and feel good?**

**Merchandise for health and well-being?**

**[Knowledge] What do you think about, read and talk about regarding ...**

drinking, drugs, health products, skin care and beauty products?

**[Behaviour] Do you use ...**

drugs, alcohol, health products, skin care and beauty products?

**[Attitudes] What do you think about...**

drugs, pharmaceuticals, health products, skin care and beauty products

does it work?

**Focus Group 2:** Media and sources of knowledge - information about health issues among adolescents

**Knowledge - what do you know?**

**[Structure]**

What sources of knowledge and the media for information about health and that well-being are you most familiar with?

**[Process]**

What kind of information are you most familiar with in these sources and media?

**Behaviour - how do you use them?**

**[Structure]**

Which media and information sources for information on health and well-being do you use?

To what extent?

**[Process]**

How do you use available information in food choices?

How do you use available information in the choice of training?

How do you use the available information in choice of health care products, pharmaceuticals, etc.?

What impact? Coincidences / tradition / culture
(family, friends, variety, taste, knowledge, warnings, values)?

**Attitudes - what do you think of these?**

**[Structure]**

What do you think of these sources of knowledge and media? (What is credible, what is good)

What sources of knowledge would you like to exist? Is something missing? What?

**[Process]**

What do you think of the amount of information in the media you talking about?

What determines what is good / what is needed?
